# Effect of beaver dam analogs (BDAs) on waterborne protozoal pathogens *Giardia duodenalis* and *Cryptosporidium parvum*

**DOI:** 10.1128/aem.01569-24

**Published:** 2025-03-24

**Authors:** Ariel I. Loredo, Andrea Packham, Nick Graham, Skylar Johnson, Stephanie Elliott, Brian H. Bird, Carrie Monohan, Sarah Yarnell, Woutrina A. Smith

**Affiliations:** 1 The One Health Institute, School of Veterinary Medicine, University of California 70733 , Davis, California, USA; 2 The Sierra Fund, Nevada City, California, USA; 3 Department of Earth and Environmental Sciences, California State University, Chico 14663 , Chico, California, USA; 4 Center for Watershed Sciences, University of California 8789 , Davis, California, USA; UMR Processus Infectieux en Milieu Insulaire Tropical, Ste. Clotilde, France

**Keywords:** beaver dam analogs, protozoa, *Cryptosporidium parvum*, riparian wet meadow, hydrology, *Giardia lamblia*, *G*. *intestinalis*, *G*. *duodenalis*

## Abstract

Installing beaver dam analogs (BDAs) in freshwater ecosystems is a process-based restoration technique mimicking natural beaver dams to increase stream channel complexity, floodplain connectivity, and hydrological residence time. BDAs have been applied in recent years by resource managers to improve riparian habitats with beneficial effects on ecosystem services, including improved water quality. While BDAs have been shown to reduce suspended sediments and nutrients in surface waters, research is needed to evaluate BDA effects on waterborne fecal pathogens such as *Giardia duodenalis* (syn. *G. lamblia*, *G. intestinalis*) and *Cryptosporidium parvum*. At a California riparian field site, this study used an *in situ* release trial of inactivated protozoal (oo)cysts upstream of BDAs to compare the concentrations and loads detected above and below the BDAs. The median percent decrease in load-based recovery rates was 78% for *G. duodenalis* and 80% for *C. parvum* at sites with BDAs. Mixed-effect linear regression analyses showed a significant reduction (by 81%) of the *G. duodenalis* adjusted cyst concentrations in downstream surface waters at sites with BDAs compared to similar sites without BDAs. While a reduction of *C. parvum* oocysts was noted, it was not statistically significant. The improved surface water quality downstream of BDAs could be because the BDA structures promote hyporheic exchange and act as a passive filter to remove pathogenic protozoa from streams. If BDAs are used to aid the restoration of riparian meadow ecosystems to slow water and raise the water table, they may also help to promote cleaner and safer waters for communities downstream.

**IMPORTANCE:**

Beaver dam analogs (BDAs) are a cost-effective, low-environmental impact technique for stream and riparian meadow restoration that provide a variety of beneficial ecosystem services; however, their impact on waterborne fecal protozoal pathogens has not been evaluated. This study used an *in situ* protozoal release trial to quantify the effect of BDAs on the load of *Giardia duodenalis* and *Cryptosporidium parvum* in streams in California. Results showed that *G. duodenalis* concentrations below BDAs were significantly reduced by 81%. The median percent decrease in load-based recovery rates below a BDA for *G. duodenalis* and *C. parvum* was 78% and 80%, respectively. This finding indicates that BDAs may promote passive filtration of waterborne pathogens, thereby improving water quality in downstream reaches and adding to the beneficial outcomes associated with using BDAs in stream and meadow restoration efforts. The potential benefit to resource managers and communities is immense.

## INTRODUCTION

Beaver dam analogs (BDAs) are increasingly being used as a method of process-based restoration that rapidly enhances riparian stream-floodplain interactions and can result in increased channel complexity with comparable overall ecosystem benefits to natural beaver dams (1–6). As the name suggests, constructing a BDA involves mimicking natural beaver dams by creating vegetation-based structures to slow down surface waters, and they are relatively low cost compared to other habitat interventions such as rock check dams. BDAs are built using various methods, including weaving riparian vegetation through in-stream vertical posts or packing natural channel materials behind vegetation that is wedged between in-stream vertical posts (6). BDAs function predominantly to slow streamflow, create ponding onto the adjacent floodplain, and promote sediment aggradation upstream, thereby spreading the surface waters out and increasing floodplain connectivity and in-stream complexity (3, 4). BDAs provide a cost-effective approach for restoring degraded stream and riparian meadow ecosystems with incised channels and dewatered adjacent floodplains. In some cases, local beavers may supplement and maintain the artificial dams over time. However, the ecological benefits to riparian meadow hydrologic function can be realized even without active beaver populations if the BDAs are maintained (4, 7).

Wet meadows are groundwater-dependent ecosystems consisting of fine-textured soils, extensive hydrologic connectivity, and prominently herbaceous plants with woody plants that can tolerate anaerobic conditions, such as willows (8). These ecosystems provide drought resistance, mitigate carbon and nitrogen emissions, and promote the biodiversity of pollinators, nesting birds, and other wildlife (8–12). Disturbances to wet meadow ecosystems, such as mechanical disruption of riparian streambanks by vehicles, people, and/or animals, can result in high rates of stream incision and bank erosion, leading to reductions in low-flow, late-season surface water availability and reductions in greenhouse gas sequestration seasonally (8, 11).

Several methods of wet meadow restoration are reported in the literature. These methods can be grouped into channel-filling restorations, appropriate for subsurface flow-dominated meadows, and in-stream structural restorations, such as BDAs, that are suitable for riparian meadows with natural stream channels (8). Over the past 20 years, BDAs have become popular as a low-environmental impact, cost-effective restoration method for streams and riparian meadows. Previous studies on the effects of BDAs generally show improvements in the abundance of riparian vegetation, increases in channel complexity, increases in carbon sequestration, decreases in summer water temperatures, and increased reconnection of the stream with its floodplain (2, 3, 13, 14).

Natural beaver dams and BDAs have also been noted to improve water quality and habitat for aquabiota (3, 7, 15–18) as both can act as a “trickle filter,” which is an enclosed bed of filter medium that encourages biofilm growth and distributes water through or over the medium (18, 19). This filtration effect has been observed for dissolved organic carbon, nitrogen, phosphorus, and suspended sediments (19, 20). However, the impact of BDAs on the transport of waterborne zoonotic fecal protozoal pathogens has not been studied.

**Fig 1:**
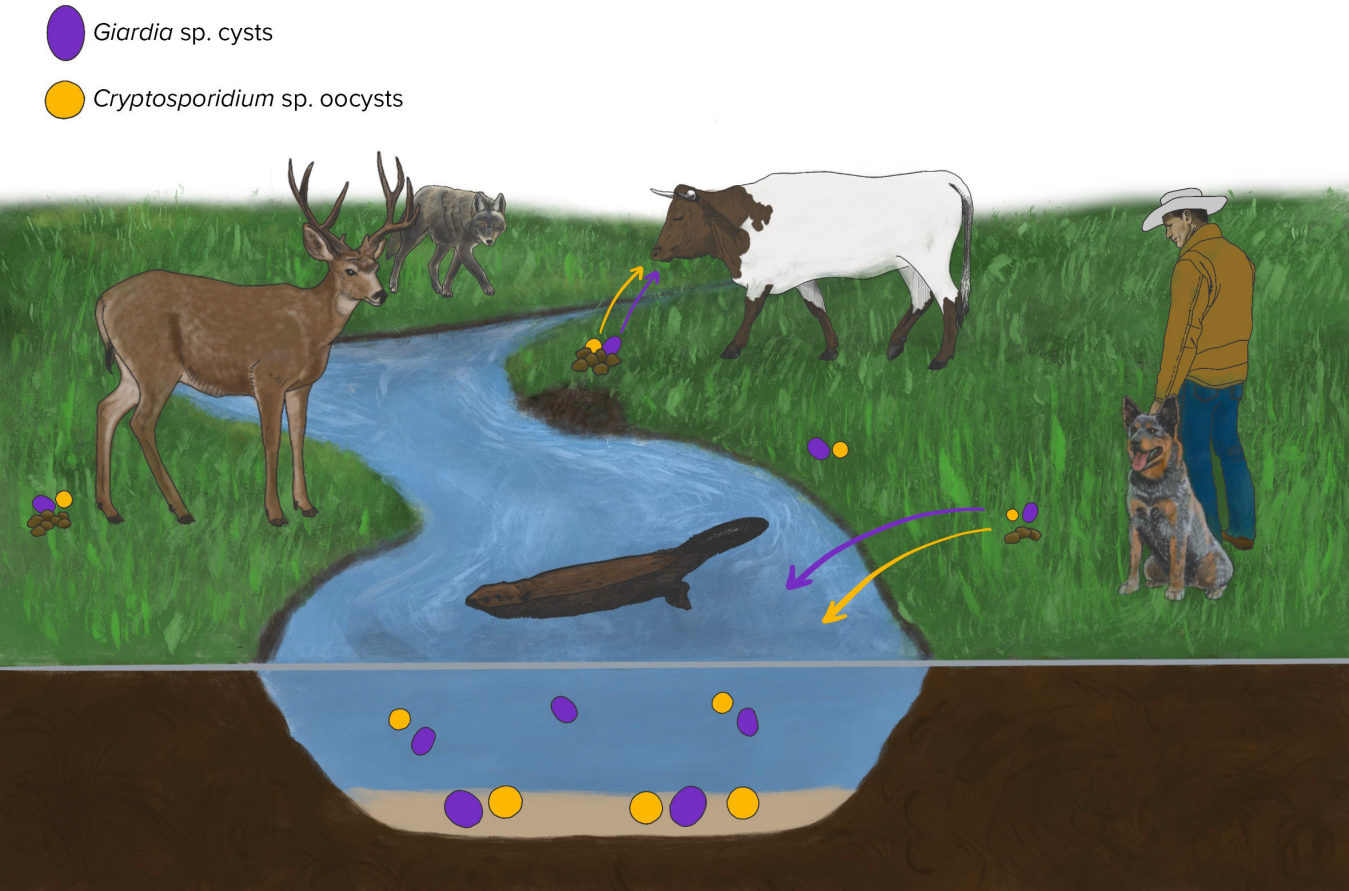
This figure represents a generalized lifecycle of *Giardia* spp. and *Cryptosporidium* spp. and their relationship to waterways. These oo(cyst)s exit the gastrointestinal tract in their infectious stage in feces. The oo(cyst)s can be directly ingested by a host or enter the waterways. They are environmentally persistent and infectious for months to years, depending on the environmental conditions. The water contaminated with the oo(cyst)s can be ingested by a host, allowing the protozoa to restart their lifecycle in the host. All the animals pictured are possible hosts that can enable the lifecycle of *Giardia* spp. or *Cryptosporidium* spp. to be completed. Image created by E. Preston, reproduced with permission.

*Giardia* and *Cryptosporidium* protozoal, zoonotic parasites that cause waterborne diarrheal disease in both humans and animals. In the United States, giardiasis and cryptosporidiosis are reportable diseases in humans. To date, cases from 2019 have been analyzed and released by the Center for Disease Control. From 2012 to 2017, there were 760 reported outbreaks of giardiasis, and incidence in 2019 averaged 5.8 cases per 100,000 persons and ranged based on geographic location (21, 22). In 2019, there were 13,979 reported cases of cryptosporidiosis in humans, having an incidence of 4.3 cases per 100,000 persons (23). Outbreaks for both diseases have been reported frequently in 2023 and 2024. These are typically associated with childcare facilities, outdoor water sports, and contact with wildlife (24–26). Figure 1 outlines the generalized environmental lifecycle of these parasites, which are shed in the host feces as an oocyst or cyst stage. These protozoa have a wide range of potential hosts, including humans, wild and domestic ungulates, avian species, wild and domestic carnivores, and reptiles. Some of the parasite species are primarily host-specific, while others have significant zoonotic health risks and can cross from animals to humans through environmental exposure (27–29). The oo(cyst)s of *Giardia* and *Cryptosporidium* spp. are environmentally persistent and have a low infectious dose (27, 28). Waterborne transmission is the most common route of infection after direct fecal-oral contact (e.g., ingestion of feces) for people and animals (27, 28).

Studies investigating the water transport properties of these fecal protozoa have noted that they settle rapidly in slower waters (30–32). The presence of vegetation in surface waters has also been found to decrease loading of fecal protozoa in downstream flows (32–35). BDAs both decrease the velocity of stream flow and increase the presence of vegetation within the channel, and therefore, we hypothesized that BDAs would be associated with reduced concentrations and loads of fecal protozoa downstream of the BDA compared to upstream.

To test this hypothesis, an *in situ* inactivated protozoal release pilot trial was performed at three BDA stream sites and a non-BDA control stream site, all located in a high mountain meadow riparian ecosystem in Plumas County, CA. We hypothesized that the recovery rates of both types of protozoal oo(cyst)s would decrease by at least 40% based on previous literature (32) after passing through an in-stream BDA compared to recovery over a similar distance in a stream site without a BDA.

## MATERIALS AND METHODS

### Field methods

**Fig 2:**
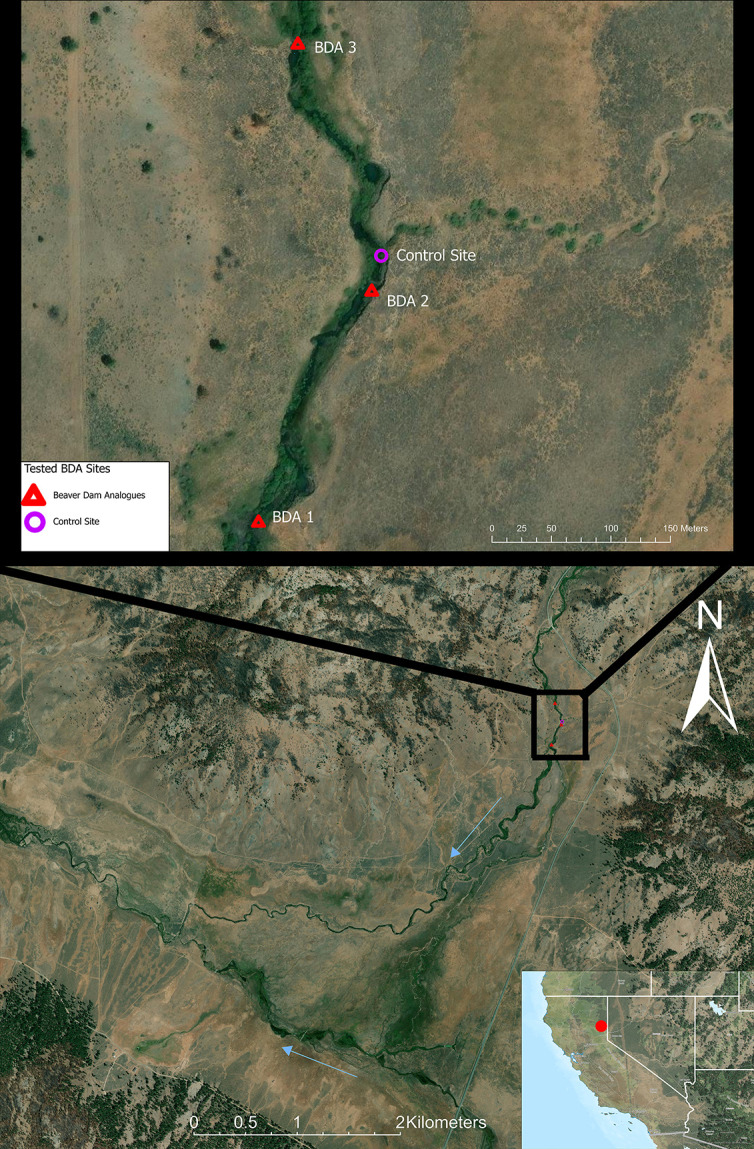
A map of the Red Clover Valley with Dixie Creek (most northernly stream) running north to west (blue arrow) and Red Clover Creek running south to west (blue arrow) before joining in the western edge of the valley in Plumas County, CA. The locations of tested BDAs (red triangles) are highlighted within the inset map above. Streamflow directionality is represented with blue arrows. BDA 3 was the most upstream, and the experimental trial occurred in the same timeframe as BDA 2. BDA 1 was the first pilot test performed in 2021 and was the most downstream. The control site (open purple dot) was located directly upstream of BDA 2. Maps were created in ArcGIS Pro 3.2.1 under University of California, Davis’s advanced license (Earthstar geographics, ESRI, USGS).

The study sites were located in a 3,000-acre multi-use riparian meadow ecosystem at 5,500 ft elevation that was undergoing meadow and stream restoration in Plumas County, CA (Fig. 2). Over the past century, the meadows and streams had experienced dramatic soil erosion and habitat degradation due to anthropogenic impacts. A diverse partner consortium implemented restoration activities to restore the riparian meadow ecosystem, including the installation of 42 BDAs across two streams that feed into the Feather River.

Three BDA study sites and one non-BDA control site were chosen within a 380 m stream reach of Dixie Creek (Fig. 2). BDA sites with limited riparian or in-stream vegetation at least 1 m upstream or downstream of the structure were selected to isolate the vegetative structure’s influence on protozoal recovery loads. BDAs selected were also examined to ensure water was not flowing through visible holes in the structure to maximize the stream water coming into full contact with the vegetative structure as it moved downstream. The control field site was chosen based on similar stream vegetative characteristics, cross-sectional area, and average discharge at the BDA sites, but with no BDA present.

**Fig 3:**
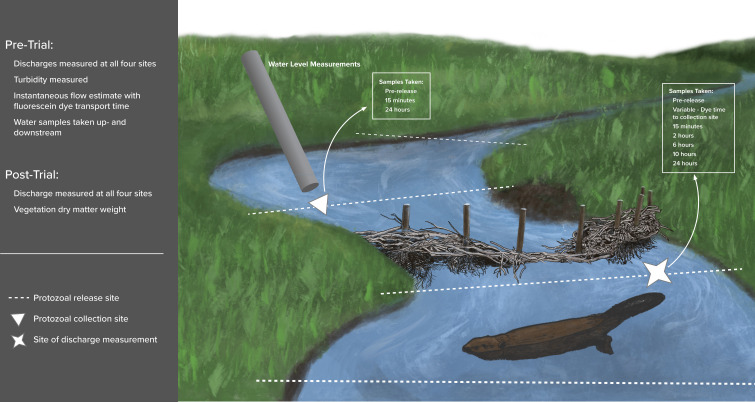
This figure outlines the general methodology used at each trial for BDA or non-BDA site recovery. Pre-trial, before protozoal release, measurements are outlined in the upper left of the figure. Post-trial, after the last sample collection, measurements are outlined in the upper right of the figure. The relative location of the vertical stilling well, site of stream discharge measurements, protozoal release, and collection site are noted in reference to the BDA site. The timing for the water samples taken at each site is noted on the figure. Image created by E. Preston, reproduced with permission.

An initial inactivated protozoal release trial was completed at BDA 1 to determine the effectiveness of the sampling methodology. Similar release trials were then performed at BDA 2, BDA 3, and the control site, as described below and in Fig. 3. During the BDA 1 trial, stream discharge measurements were taken 5 m upstream and 1 m downstream from BDA 1, at 0.5–1 m intervals using a Marsh McBirney Flomate and a topset metric wading rod (36). A Levelogger (37) was installed in a vertical stilling well at the site of discharge measurements within 5 m of BDA 1 to measure water levels at 15 minute intervals. A stage-discharge rating curve was determined from the stream discharge measurements and the recorded water levels to create a 15 minute discharge time series throughout the trial.

**Fig 4:**
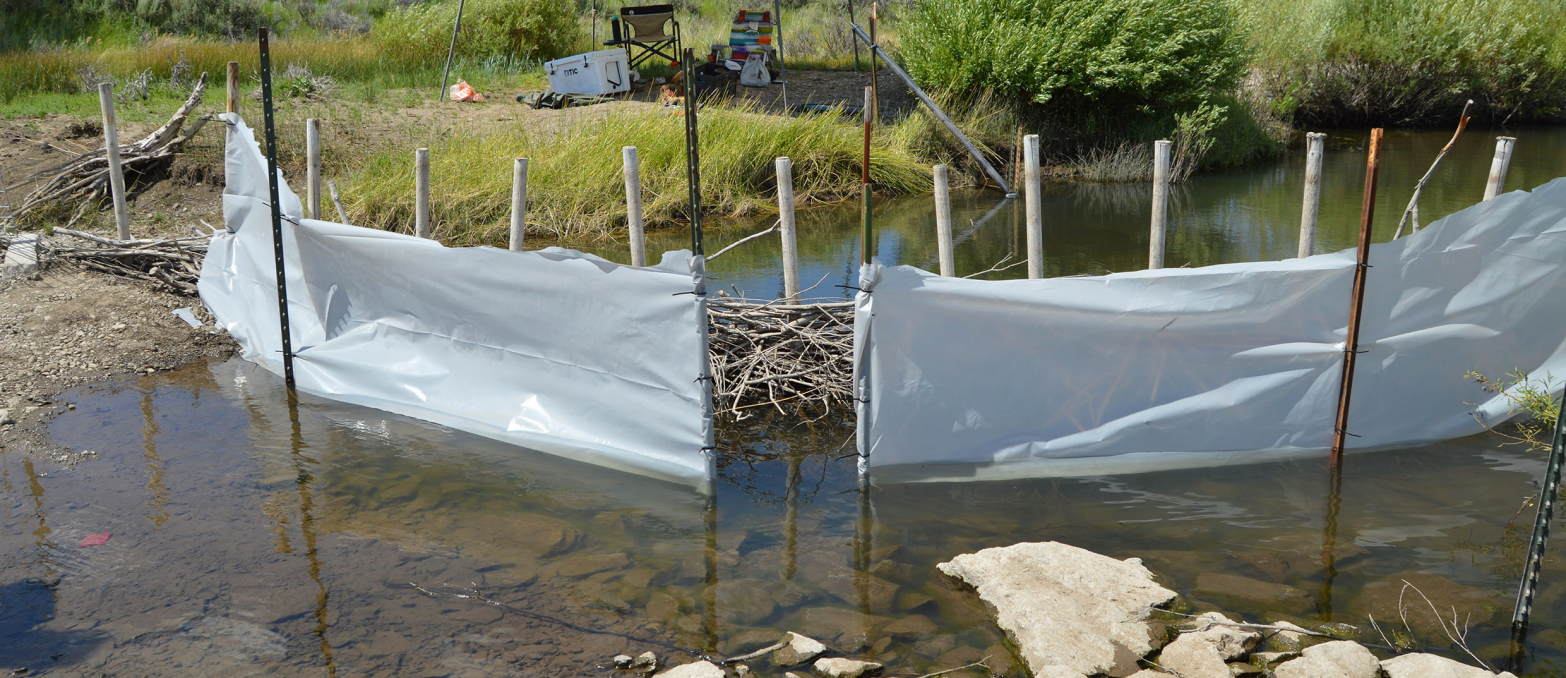
This BDA photo shows the “funnel” set up below the BDA to collect all the water in the stream flow. The gap between the funnel allows water collection. The funnel extends into 50% of the water column to avoid the impedance of the water flow. A fence post upstream marks the protozoal release site for repeatability in the replicates.

A “funnel” with an opening of 0.5 m wide was constructed of 5 mm polyethylene plastic to collect the downstream surface water below BDA 1 (Fig. 4). The funnel was placed at 50% depth in the thalweg, or the deepest point of the stream, 1 m downstream of the BDA. Samples were taken at the 0.5 m opening at the water surface. The protozoal release point was placed 1 m upstream from the middle of the BDA. At the release point, fluorescein dye (38) was released by hand directly above the surface water without the researcher disrupting the stream bed. The dye was used to estimate the expected protozoal transport time through the BDA and confirm funnel functionality. The time to first point of direct contact with BDA, time to observation of dye on the downstream side of the BDA, and time to exit the funnel at the collection point were noted. This was performed at least 12 hours before protozoal release to allow all dye to dissipate before the start of the trial. At least 1 hour before the protozoal release, 10 L water samples were taken upstream and downstream in order to establish baseline protozoal concentration. A second downstream sample was taken, and AccuSpike-IR (39) was used to create a sample spiked with a known amount of inactivated oo(cyst)s to allow adjustment for the expected rate of protozoal loss in laboratory processing. Finally, turbidity was tested using a handheld turbidimeter before the trial test to confirm that turbidity returned to pre-disturbance levels (e.g., walking in the stream) within 10 minutes of disturbing the stream bed.

**Fig 5:**
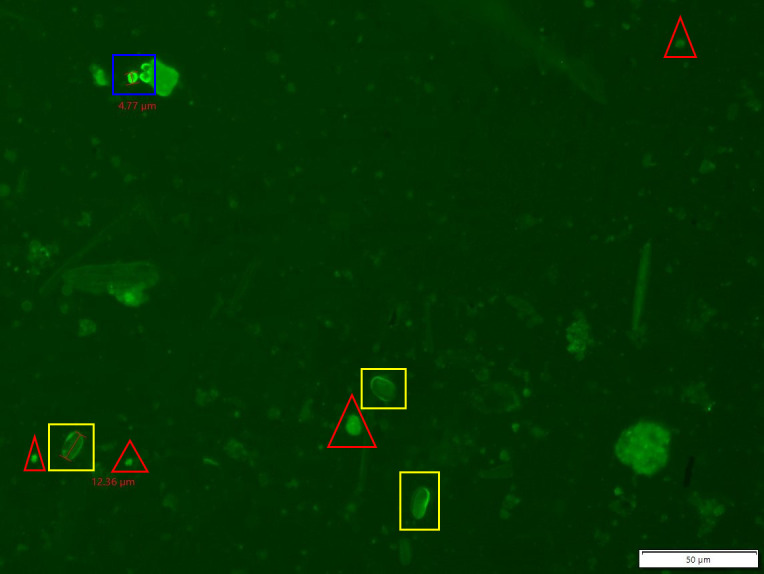
A microscopy photo of released and re-captured *Giardia lamblia* (yellow squares) and *Cryptosporidium parvum* (blue squares) dyed with immunofluorescent antibodies specific for *Giardia* sp. and *Cryptosporidium* sp. (Biopoint EasyStain). Some cross-reactions of the immunofluorescent can occur with other particulates in the water. Example particulate which might be mistaken for either *Giardia* sp. or *Cryptosporidium* sp. is noted by a red triangle. The background of other particulates in these samples was generally low, and this is a representative photo of the overall background seen throughout experimentation. (Photo credit: A. I. Loredo.)

During each release trial, 4.8 million heat-inactivated and non-infectious *Giardia duodenalis* (39) (syn. *G. lamblia*, *G. intestinalis*) and *Cryptosporidium parvum* (39) were released at the upstream release site by gently pouring the parasite solution into the creek 1 m upstream from the BDA without disturbing the stream bed (Fig. 3). Paired 10 L water samples were taken upstream and downstream of BDA 1 at 15 minutes and 24 hours post-release. Single downstream 10 L water samples were taken at 15 minutes and at 1, 2, 4, 6, 8, 18, and 24 hours post-release from the collection site downstream of BDA 1. Samples were filtered with EnviroChek HV filters (40) onsite and kept on ice during transport to the lab for processing. The laboratorians were blinded to the sample’s location and time point of collection using randomized identification numbers. Stream samples were processed per EPA Method 1623 (41) using Dynabeads GC-combo (42) and BioPoint EasyStain (43) staining kits. The spiked sample was a positive control for the filtration and immunomagnetic separation steps. The BioPoint kit positive control was used to confirm staining accuracy. The pre-experimental samples acted as endogenous negative controls. Protozoa were enumerated using fluorescent microscopy, and 10% of all slides were re-read by a second experienced protozoal diagnostic laboratorian with no significant difference (Fig. 5).

**Fig 6:**
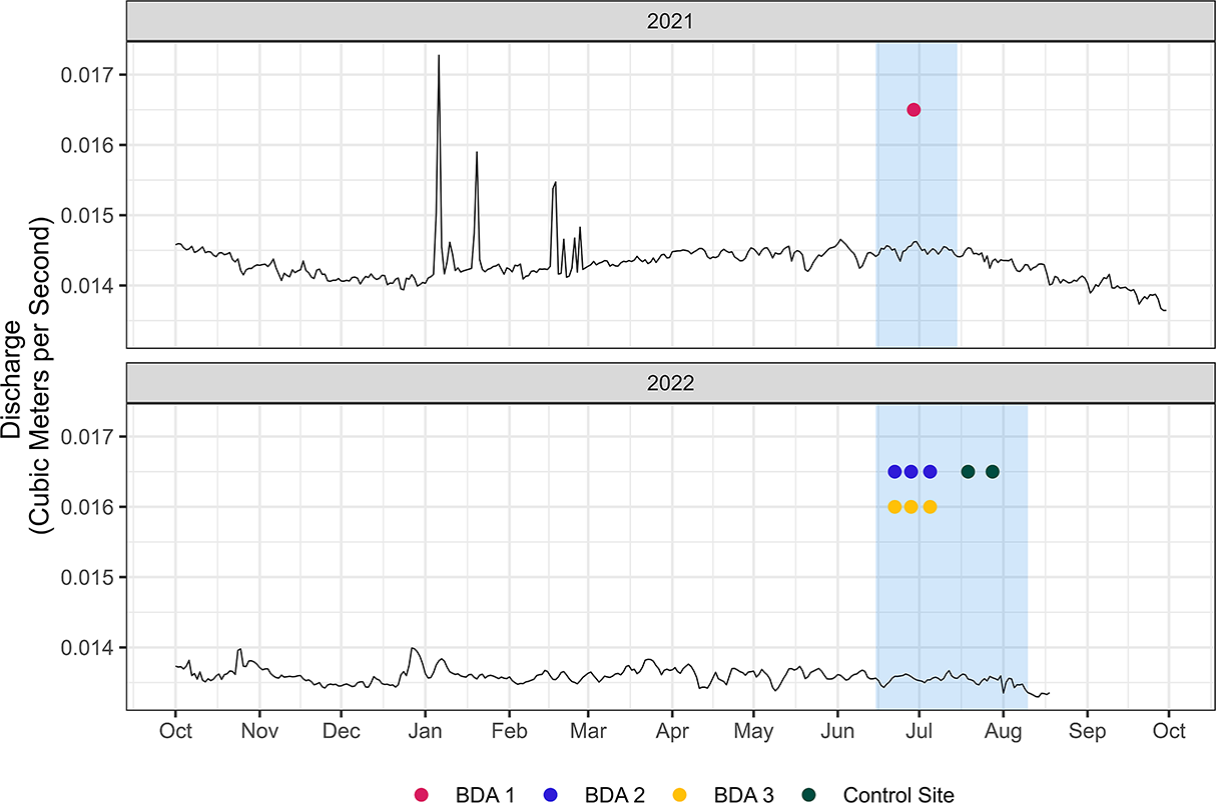
These hydrographs show the trends of annual discharge in the Dixie Creek of Clover Valley in the years 2021 and 2022. The blue highlighted regions indicated times when trials occurred on the BDAs listed. The dots indicate the 24 hour period each replicate occurred for the corresponding BDA. BDA 1 occurred in June of 2021, while BDA 2 and 3 and the control site occurred in June-July of 2022. The overall discharge in 2022 is lower than in 2021. But both years are considered low water years for this valley. This graph illustrates that the experimentation occurred at all sites during the seasonal summer baseflow that is typically seen in this region. The rating curve for this hydrograph is based on five paired measurements having an adjusted R^2^ of 0.5202. The resulting equation from this stage spanning two water years is y = 0.37348 + 0.03148×. The stage graph is shown in the supplemental materials in Fig. SB.

The same discharge, water level, turbidity measurements, protozoan release, and sampling procedures described above were used for three replicate tests at BDA 2 and BDA 3, and two replicate tests at the control site. Minor modifications were made based on results from the BDA 1 trial test, including reducing downstream sampling times to 15 minutes, 2, 6, 10, and 24 hours post-parasite release. This was based on the concentration-time curve created by the first trial, and sampling times were spaced to reflect the peak decay of this curve. Additionally, for each trial, stream discharge measurements were taken pre- and post-trial at a site further upstream and further downstream from the BDA, as well as at the levelogger within 5 m of the BDA and at the collection site below the BDA. All replicates were performed in consecutive weeks, with the BDA 2 and BDA 3 trials performed in the same weeks (Fig. 6).

After all replicate trials were completed, the in-stream vegetation was collected, and dry matter weight was measured to help evaluate the relationship between in-stream vegetation and protozoal transport. Following the first replicate trials at BDA 2 and 3, which were performed simultaneously, it was noted that the peak of the oo(cyst) flux varied with stream flow rate through each structure, which might lead to an underestimate of oo(cyst) recovery with the established collection time points. An additional downstream sample collection was added for the second and third replicates of BDA 2, 3, and all the control site replicates. The timing of the added sample for each replicate trial was determined from the dye study time from the release site to the collection site for that replicate (Fig. 3).

### Data analysis

The levelogger data were compensated for regional barometric fluctuations using a local weather station (Wunderground Station “KCACHILC7”) and used with the stage-discharge rating curves to calculate a 15 minute time series of discharges during the study. The discharge time series associated with BDA 3 was estimated based on the relationship between the measured discharges of BDA 3 and BDA 2. To place the discharges observed during the study into a seasonal context, the annual hydrograph for Dixie Creek (Fig. 6) was estimated based on a rating curve between the measured discharges in Dixie Creek and measured water levels in a nearby gaged stream, Red Clover Creek. As complete annual water levels were not available for Dixie Creek, Red Clover Creek was chosen as a comparison point for Dixie Creek due to its proximity—both run parallel in the same valley—and similar water level fluctuations (Fig. 2).

Protozoal counts in measured volumes of stream water were recorded during fluorescent microscopy analysis and led to the estimates of protozoal concentrations. During data analysis, concentrations were adjusted to account for loss during processing based on the average experimental recovery of lab-spiked protozoa for all BDAs (41). The protozoal results were plotted over time in two ways: an adjusted protozoal concentration and an adjusted protozoal load. The equation used to calculate adjusted protozoal concentration was oo(cyst) concentration divided by the average recovery rate of the spiked samples throughout the study (1.53% and 5.25% for *G. duodenalis* and *C. parvum*, respectively). Similarly, the adjusted protozoal load was calculated by the adjusted oo(cyst) concentration multiplied by the total water discharge at the time of sample collection. Using R Studio (44), a plot for each replicate trial by protozoa was created using adjusted concentration, load, and collection timepoint. The area under the curve (DescTools package) for all replicate trials was used to estimate the total recovered protozoa in each trial. The median percent difference was calculated using the following equation to weight the BDA against the control $\frac{{RR}_{control}-{RR}_{BDA}}{{RR}_{control}}x100.$ Mixed effects linear regression was used to determine the relationship between protozoal recovery rate at downstream sampling points, and considering the presence or absence of a BDA. Data met assumptions of mixed linear effects, including normality evaluated through plot assessments and residuals. R Studio was used for all analysis and figures, including the base R and “ggplot2” packages.

## RESULTS

### Characteristics of study sites

No or minimal vegetation was present at all sites in the main stream channel (Supplemental materials; Fig. SA). BDA 2 and 3 had 132 and 41 g of dry vegetation matter (Supplemental materials, Table A), respectively, in the water flow during experimentation. BDAs varied in length from 5.3 to 12.2 m. Weather conditions were generally sunny, with no precipitation during or between experimentation periods for all sites. The control site was upstream of BDA 2 and downstream of BDA 3 and had a similar discharge and channel slope to BDA 2. It also has a similar cross-sectional area to BDA 3. The spatial relationship of all these sites is outlined in Fig. 3.

The characteristics measured at each test site during the study are provided in Table 1. Additional characteristics such as temperature, turbidity, and measured length of BDA are provided in supplemental materials in Table SA. BDA 1 trials were conducted in June 2021, BDA 2 and 3 were sampled in June to July 2022, and the control site was sampled in July 2022 (Fig. 5). All three BDAs had a similar stream discharge during the trials, while the control site had a lower stream discharge. Calculated water discharges declined slowly over time between the replicate trials at BDA 2, BDA 3, and the control site. BDA 1 and the control site had the lowest discharge of <0.01 m^3^/s, while discharge at the other two BDAs was 0.044 and 0.05 m^3^/s.

**TABLE 1:** Descriptive statistics of the measured characteristics between the study sites

	Beaver dam analog sites			Control site
	BDA 1	BDA 2	BDA 3	
Mean water discharge (m^3^/s)				
Median (IQR)	0.019 (–)^*a*^	0.046 (0.038, 0.066)	0.052 (0.036, 0.061)	0.017 (0.009, 0.026)
Range	–	0.030, 0.086	0.020, 0.069	0.000, 0.035
Rating curve statistics				
R-squared	1	0.6738	0.8921 (vs BD2)	0.7108
Number of measurements contributing to the stage	2	11	9	8
Timeframe of measurements (weeks)	1 June 2021	3 June to July 2022	3 June to July 2022	2 July 2022

^*a*^ – indicates no numerical value.

Figure 6 shows the annual hydrographs for Dixie Creek in 2021 and 2022 based on a rating curve determined from manually measured discharges in Dixie Creek, just downstream of the study sites, and water level measurements from a levellogger in nearby Red Clover Valley Creek. Time periods when this study occurred are highlighted in blue, showing that experimentation on BDA 2, 3, and the control site occurred at a similar summer baseflow.

### *Giardia duodenalis* recovery rates below BDAs

The downstream recovery rates for *G. duodenalis* as an adjusted concentration ranged from 0.02% to 0.15% across all BDA sites and replicates (*n* = 7) with a median of 0.03% between sites and replicates (Table 2). The median recovery rate at the downstream control site was 0.27% (*n* = 2), ranging from 0.19% to 0.35%. This equates to a median percent decrease ranging from 81% to 93% at each individual BDA site as compared to the control site. Collectively, across all BDA sites, there was a median percent decrease of 91% of *G. duodenalis* as compared to the control site that did not have a BDA present between the upstream and downstream sampling sites.

**TABLE 2:** Summary characteristics for adjusted concentration and load-based recovery rates of *Giardia duodenalis* and *Cryptosporidium parvum* in surface waters

	Beaver dam analog sites			Control site
	BDA 1 1 replicate	BDA 2 3 replicates	BDA 3 3 replicates	2 replicates
*G. duodenalis* adjusted concentration recovery rate (%)				
Median (IQR)	0.02 (–)^*a*^	0.02 (0.02, 0.05)	0.05 (0.04, 0.10)	0.27 (0.23, 0.31)
Range	–	0.01, 0.07	0.02, 0.15	0.19, 0.35
Median percent decrease to comparison	93%	93%	81%	–
*G. duodenalis* load recovery rate (%)				
Median (IQR)	0.67 (–)	2.09 (1.43, 2.61)	1.61 (1.59, 2.13)	7.21 (3.94, 10.49)
Range	–	0.77, 3.13	1.58, 2.64	0.66, 13.76
Median percent decrease to comparison	91%	71%	78%	–
*C. parvum* adjusted concentration recovery rate (%)				
Median (IQR)	0.01 (–)	0.01 (0.01, 0.12)	0.04 (0.02, 0.06)	0.13 (0.11, 0.16)
Range	–	0.01, 0.24	0.00, 0.08	0.08, 0.19
Median percent decrease to comparison	92%	92%	69%	–
*C. parvum* load recovery rate (%)				
Median (IQR)	0.31 (–)	1.10 (0.82, 6.09)	0.66 (0.47, 1.50)	3.31 (1.84, 4.77)
Range	–	0.55, 11.09	0.29, 2.34	0.38, 6.24
Median percent decrease to comparison	91%	67%	80%	–

^*a*^ – indicates no numerical value.

**Fig 7:**
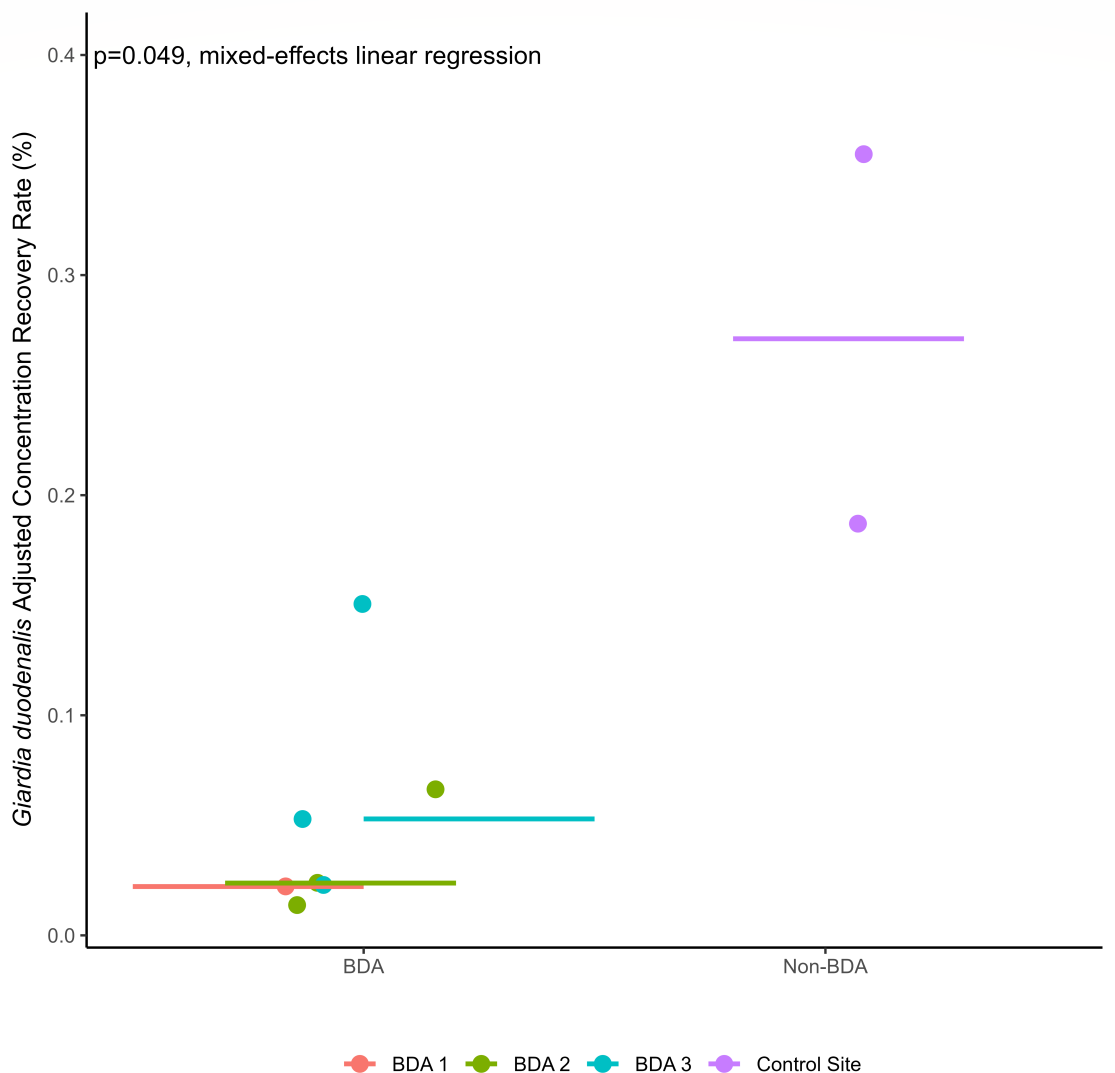
This dot plot shows the recovery rates for *G. duodenalis* at all four sites. The horizontal line indicates the median recovery rate for each site. The purple color on the right shows the non-BDA site’s wide range of recovery rates. The left side of the plot shows the breakdown at the three BDA sites. The BDA sites had an 81% decrease in recovery rates as compared to the non-BDA control site.

When considered as load-based recovery rates, 0.67%–3.13% of the cyst load was recovered downstream across the BDA sites and replicates (*n* = 7). At the control site, 0.66% and 13.76% recovery rates were seen downstream. This is a median percent decrease of 78% (71%–91%) between BDA sites as compared to the control site without a BDA (see Fig. 7). These results are summarized in Table 2. Considering the downstream recovery rates for *G. duodenalis* as adjusted cyst concentrations, recovery ranged from 0.02% to 0.15% across all BDA sites and replicates (*n* = 7), with a median of 0.03% between sites and replicates (Table 2). The median cyst recovery rate at the downstream control site was 0.27% (*n* = 2), ranging from 0.19% to 0.35%. This suggests a median percent decrease ranging from 81% to 93% at each individual BDA site as compared to the control site. Collectively, across all BDA sites, there was a median percent decrease of 91% of *G. duodenalis* as compared to the control site that did not have a BDA present between the upstream and downstream sampling sites.

The relationship between the adjusted concentration-based recovery rates of *G. duodenalis* at all BDAs and the non-BDA site is shown in Fig. 7. This plot shows the distribution of the recovery rates at the BDA sites and control sites. When this relationship was controlled for repeated sampling, a statistically significant difference was found for *G. duodenalis*-adjusted concentration between a site with a BDA and without a BDA (mixed-effects linear regression, *P* = 0.049). This model predicts a reduction in BDA recovery rates by 0.22%, which is an 81% decrease as compared to the predicted recovery rates in the control site (Table 2). This significance was not found when *G. duodenalis* was considered as a load-based recovery rate (*P* = 0.20).

### *Cryptosporidium parvum* recovery rates below BDAs

**Fig 8:**
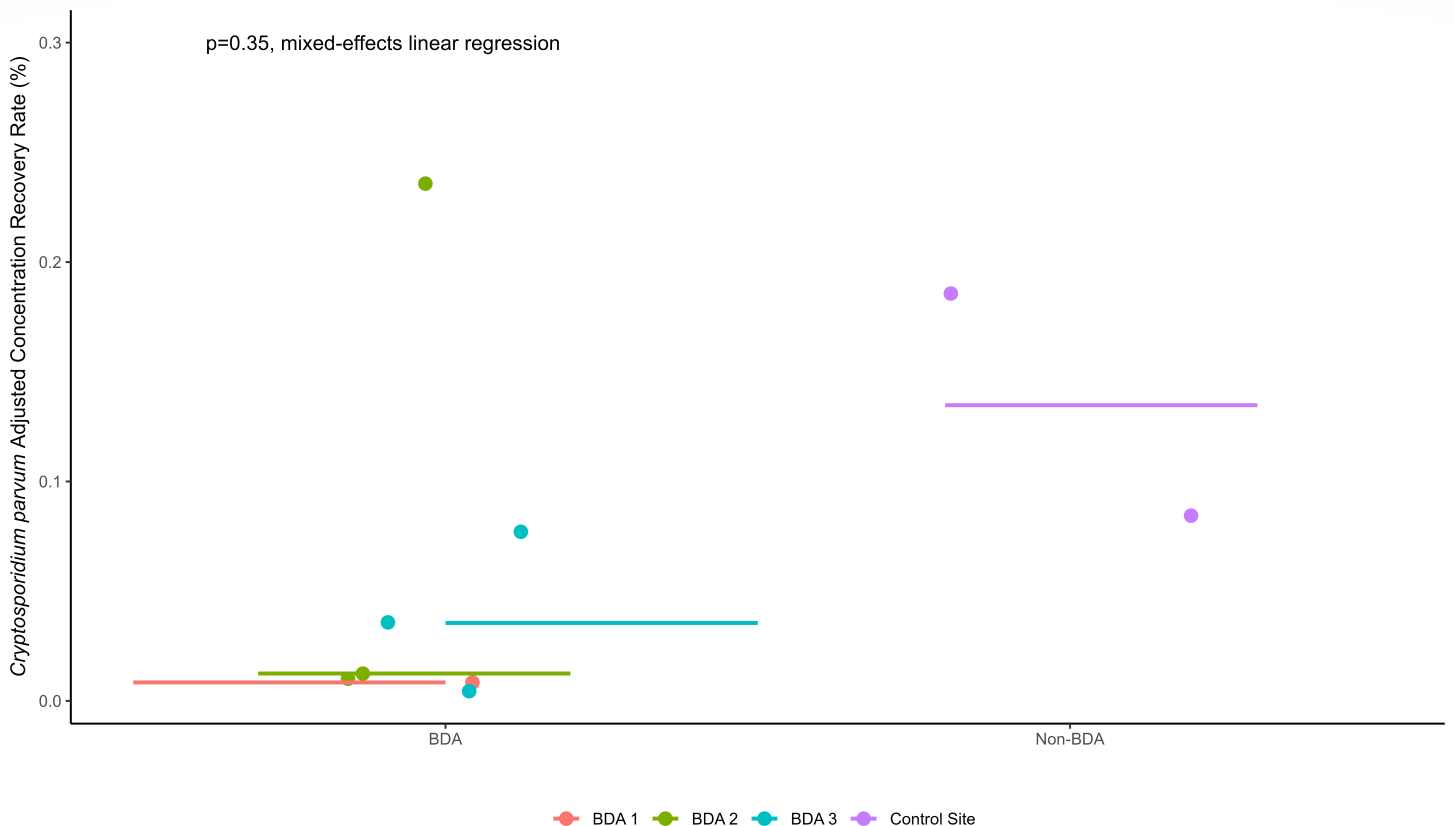
This dot plot shows the concentration-based recovery rates of *C. parvum* at each sampling site. The horizontal line indicates the median recovery rate for each site. The purple boxplot shows the non-BDA site, while the boxplots on the left side of the plot show the BDA sites. The BDA sites have a lower recovery rate than the non-BDA sites, with a 90.7% median percent decrease.

When considered as load-based recovery rates, *C. parvum* recovery downstream ranged from 0.29% to 11.09% at the BDA field sites, while recovery rates of 0.38%–6.24% occurred at the control site, resulting in a median percent decrease of 67%–91% at the BDA sites compared to the control site. Collectively, across all BDA sites (*n* = 7), there was a median percent decrease of 80% for *C. parvum* compared to the control site (*n* = 2, Fig. 8). The recovery rates for *C. parvum* are listed in Table 2. When considered as an adjusted concentration-based recovery rate, 0.01%–0.24% of *C. parvum* oocysts were recovered downstream from the BDA sites, with a median recovery rate of 0.35% (*n* = 7, Table 2). For the control stream site, downstream recovery rates ranged from 0.08% to 0.19%, with a median of 0.13% (*n* = 2). Thus, the median percent decrease of a concentration-based *C. parvum* recovery rate at the individual BDAs compared to the control site ranged from 69% to 92%. Figure 8 shows the distribution of the adjusted concentration-based recovery rates of *C. parvum* at the BDA and non-BDA sites.

There was higher variability in the recovery rates of *C. parvum* compared to the recovery rates seen for *G. duodenalis*. Using mixed-effects linear regression, the difference in adjusted concentration and load-based recovery rates of *C. parvum* at the BDA and non-BDA sites lacked statistical significance (*P* = 0.35, 0.79, respectively).

## DISCUSSION

This study was a significant step forward in understanding the effect of BDAs as filters for *G. duodenalis* and *C. parvum* in the surface water of a high-elevation wet meadow ecosystem in the Sierra Nevada mountains. The results were promising, indicating that the transit of protozoa through BDAs could reduce the adjusted concentration of *G. duodenalis* in the surface water of this riparian stream system by 81% compared to sites without a BDA. Although the case *of C. parvum* didn't yield statistically significant results, the numerical trend suggested a load reduction of 80% compared to sites without a BDA. These findings hint at the potential of BDAs in reducing the overall adjusted concentration of *G. duodenalis* in stream surface waters, compared to sites without a BDA in place across the stream. The implications are that health risks are reduced if BDAs are in place to improve water quality by reducing downstream transport of fecal pathogens from wildlife (e.g., beaver fever), domestic animals, and humans. However, further studies are needed to fully understand the overall impact of protozoal load for both *G. duodenalis* and *C. parvum*. The positive environmental impact of BDAs has been well-documented (1–4, 7, 9, 13, 15–17, 19, 20). This study further enriches our understanding of the ecosystem service benefits BDAs offer in wet meadow ecosystems (19, 20) as trickle filters for *G. duodenalis*.

This study also found a difference in the measurable effects of the BDA on *C. parvum* and *G. duodenalis* parasites. When either measurement of *C. parvum* was considered, no statistically significant difference was found between the control and BDA sites. However, the BDA effect on the adjusted concentration of *G. duodenalis* in stream water samples was found to be statistically significant. The control site recovery rates varied widely, perhaps due to the changes in stream eddies described above. That variation is magnified by accounting for the volume of water at the site through the load measurement, which influences the lack of statistical significance seen. This highlights two important features: the overall impact of water flow on protozoal dynamics and the properties of protozoal adherence. *C. parvum* is more hydrophilic in nature than *G. duodenalis* and, thus, is more likely to be removed from vegetation with increased flow (45). Other studies also suggest that *C. parvum* is not as highly influenced by the presence of vegetation in the streamflow as *G. duodenalis* (32). These findings suggest that load-based recovery rates might be a more environmentally accurate measurement of protozoa. Also, the literature suggests that understanding protozoal dynamics is a vital interplay between each protozoa’s unique vegetation adherence properties and stream dynamics. As the numerical direction of all protozoal outcomes suggests a reduction in recovery rates, it provides evidence that BDAs might act as trickle filters for both protozoa if we can gain sufficient statistical resolution.

The underlying mechanisms of how the BDAs might act as trickle filters for these protozoa are suggested to be predominantly due to adherence to vegetation or mechanical filtering. The effects of vegetation on improving surface water quality by reducing *G. duodenalis* and *C. parvum* have been observed in previous studies. For example, on coastal California cattle farms, it was shown that planting vegetated buffers between cattle and riparian waterways slowed storm runoff from dairy farms, and that for each meter of vegetated buffer length, there was a significant reduction of protozoal pathogen loading during storm events (33, 35). Another study in coastal Californian wetlands (32) used mesocosm tank protozoal release experiments evaluating multiple factors, including the presence of vegetation within the water flow. This study found a marked reduction of over half of released *G. duodenalis* cysts when bulrushes were present in the water. In Germany, researchers found that constructed wetland wastewater plants can have a 2-log reduction in *Cryptosporidium* and *Giardia* sp. oo(cyst)s, and these findings were largely attributed to the mechanical action of small particle filter beds (46). The interwoven structure of the BDAs might act as a similar filter for the fecal protozoal pathogens in streams.

An alternative or symbiotic mechanism of action for protozoal reduction from the BDAs is the action of hyporheic flow. The hyporheic zone is the stream’s underlying shallow mixing and storage region, providing rapid exchange of surface and subsurface water, including the associated gases, microorganisms, and pollutants (47, 48). The presence of BDAs has been shown to increase vertical fluxes of the stream and can promote increased hyporheic exchange (3, 49). One study modeled that the accumulation of *Cryptosporidium* sp. in the hyporheic zone can account for up to 66% of in-stream decreases in the oocysts (50). This suggests that the hyporheic zone may also contribute to the effects seen in this study, providing a testable hypothesis for future work.

Another consideration that could contribute to removing *C. parvum* and *G. duodenalis* in surface waters passing through BDAs is the sedimentation of oo(cysts). Reinoso et al. (51) found that constructed subsurface flow wetlands removed more than 97% of the oo(cyst)s present. The facultative pond in their study was the second-highest contributor to *C. parvum* reduction (51). Several studies have also established the role of sedimentation in wetlands for removing pollutants and pathogens (52–54). For this BDA pilot study, considering the slow settling rate of these oo(cyst)s (45), the 2-m sampling length, and the relatively low turbidity of the streams (52), the role of sedimentation in the removal of these protozoa from the surface water is considered minimal. However, it should be considered a possible mechanism in the second control replicate due to the circular eddies, lower discharge, and lower recovery rates seen.

This study also identified several areas of interest for future studies. These could include increasing the number of intervention and control sites evaluated, pairing field with mesocosm tank studies, use of viable oo(cyst)s, and broader parameter inclusion to represent the complexity of the field sites. The control site in this study was chosen to mimic the hydrology and behavior of the stream when a BDA was not present. However, in this study, the control site was found to have diurnal changes in discharges, leading to a difference in average discharges between control and BDA sites. Increasing the number of control sites would provide a broader comparison group to evaluate our hypotheses. Similarly, mesocosm tank studies could help to determine the effect size of the different mechanisms by which BDAs may filter protozoa and standardize for factors such as discharge, stream dynamics, and presence of other organisms (e.g., pollens, yeast) that can cross-react and visually be confused for *C. parvum*. This cross-reaction can lead to an overestimation of *C. parvum*. It will also allow for a wholly lab-based protozoal recovery methodology, which, in these authors’ experience, has resulted in higher recovery rates for spiked water samples using the same streams. While an *ex situ* study cannot replace the importance of an *in situ* study, it may help to find greater precision in the measurements in this complex ecosystem. Similarly, inactivated oo(cyst)s may not behave identically to viable oo(cyst)s. One study suggests higher deposition rates and adherence than viable oo(cyst)s (55). Use of mesocosm tanks would allow use of viable protozoa without contamination of the environment. In future studies, the inclusion of BDAs of different construction types and ages would help to understand changes in filtration ability over time.

In summary, this study’s findings suggest that BDAs are a promising and cost-effective method to restore degraded riparian wet meadows and may have desired impacts in reducing some fecal pathogen pollution of the surface waters. Based on this pilot *in situ* experimental method and literature-based support, it is suggested that BDAs may act as a trickle filter for *G. duodenalis* in a riparian ecosystem. This simple intervention provides essential ecosystem services that can lead to drought resistance and provide cleaner surface waters. Associated downstream effects can improve the public health of wildlife and humans while enhancing the ecosystem’s health and resilience from the anthropogenic and climate change impacts on this environment with small steps.

## SUPPLEMENTAL MATERIAL

Supplemental Fig. A aem.01569-24-s0001.tif Photos of BDA 1, BDA 2, BDA 3, and control site.

Supplemental Fig. B aem.01569-24-s0002.tif Stage graph used to create the annual hydrograph using water pressures from Red Clover Creek and the manual discharges for Dixie Creek.

Supplemental material aem.01569-24-s0003.docx Supplemental Table A and legends for supplemental figures.

ASM does not own the copyrights to Supplemental Material that may be linked to, or accessed through, an article. The authors have granted ASM a non-exclusive, world-wide license to publish the Supplemental Material files. Please contact the corresponding author directly for reuse.
