# Supplementary material for: Effect of beaver dam analogs (BDAs) on waterborne protozoal pathogens Giardia duodenalis and Cryptosporidium parvum
Source: Appl Environ Microbiol. 2025 Mar 24;91(4):e01569-24. doi: 10.1128/aem.01569-24 (PMC12016503; doi:10.1128/aem.01569-24)
Supplement: Supplemental material — Supplemental Table A and legends for supplemental figures. [file aem.01569-24-s0003.docx]

**Supplemental Materials:**

| Table A: Descriptive statistics of the measured characteristics between the study sites | | | | |
| --- | --- | --- | --- | --- |
|  | Beaver Dam Analogue Sites | | | Control Site |
|  | BDA 1 | BDA 2 | BDA 3 |  |
| **Max Water Discharge (m^3^/s)** |  |  |  |  |
| Median (IQR) | 0.047 (--) | 0.052 (0.043, 0.072) | 0.059 (0.044, 0.068) | 0.025 (0.015, 0.035) |
| Range | -- | 0.035, 0.092 | 0.029, 0.078 | 0.005, 0.045 |
| **Min Water Discharge (m^3^/s)** |  |  |  |  |
| Median (IQR) | 0.004 (--) | 0.044 (0.035, 0.063) | 0.050 (0.033, 0.056) | 0.005 (-0.003, 0.014) |
| Range | -- | 0.026, 0.082 | 0.017, 0.063 | -0.011, 0.022 |
| **Max Water Temperature (°C)** |  |  |  |  |
| Median (IQR) | 26.09 (--) | 19.50 (19.05, 19.69) | 18.97 (18.30, 19.88) | 21.10 (20.75, 21.45) |
| Range | -- | 18.60, 19.88 | 17.63, 20.80 | 20.40, 21.80 |
| **Mean Water Temperature (°C)** |  |  |  |  |
| Median (IQR) | 23.59 (--) | 15.57 (15.55, 16.02) | 15.26 (15.21, 15.94) | 17.42 (17.30, 17.54) |
| Range | (--) | 15.53, 16.47 | 15.15, 16.63 | 17.17, 17.66 |
| **Min Water Temperature (°C)** |  |  |  |  |
| Median (IQR) | 20.30 (--) | 14.70 (14.25, 15.03) | 14.56 (14.07, 15.03) | 16.25 (16.22, 16.28) |
| Range | -- | 13.80, 15.37 | 13.58, 15.50 | 16.20, 16.30 |
| **Dry Weight of Vegetation within funnel (g)** | 0 | 139 | 41 | 0 |
| **Pre-disturbance Turbidity (NTU)** | -- | 4.9 | 2.10 | 4.30 |
| **Length of BDA (bank to bank, m)** | 5.3 | 5.5 | 12.2 | NA |

Supplemental *Figure A: A - BDA 1– This is a simple BDA with a wide pond behind it. There is no vegetation between the release and collection site. The cattle guard was removed for the trial. – BDA 2 – This BDA is the furthest downstream of the BDAs with three replicates. It has the most vegetation of all the BDAs (132 g dry weight). There is a slow-moving pond upstream of the BDA, which then has a small shelf of ~1 meter in distance before the water enters the BDA. C – BDA 3 – This BDA has a topology upstream similar to that of BDA 2. It is the most upstream of all the sites. D – Control site – this site is upstream from BDA 2 and downstream from BDA 3. It is a narrower area with slow-moving, deep-water column. At the time of BDA testing, the measured discharge in this area was equal to that of the BDAs.*

Supplemental Figure B: This figure shows the stage graph used to create the annual hydrograph using water pressures from Red Clover Creek and the manual discharges for Dixie Creek. The resulting adjusted R^2^ was 0.5202
